# Microelectrode Voltammetric Analysis of Low Concentrations of Se(IV) Ions in Environmental Waters

**DOI:** 10.3390/molecules29071583

**Published:** 2024-04-02

**Authors:** Malgorzata Grabarczyk, Marzena Fialek

**Affiliations:** Department of Analytical Chemistry, Institute of Chemical Sciences, Faculty of Chemistry, Maria Curie-Sklodowska University, 20-031 Lublin, Poland; malgorzata.grabarczyk@mail.umcs.pl

**Keywords:** solid bismuth microelectrode, differential pulse cathodic stripping voltammetry, eco-friendly detection of selenium, environmental water samples

## Abstract

The current research is an attempt to analyze on-site selenium(IV) ions in environmental water samples using an eco-friendly miniaturized sensor developed by deposition of a very thin amount of metallic bismuth in a solid Bi electrode tightly closed in miniaturized housing. Numerous experimental variables are optimized, including the composition of the supporting electrolyte and its pH, as well as activation and accumulation conditions. Under optimized measurement conditions, the method shows high sensitivity, permitting a very low limit of detection equal to 7 × 10^−10^ mol L^−1^ to be achieved in a short accumulation time of 50 s. The performance of this microsensor was investigated against numerous interference factors and its good anti-interference capability was demonstrated. A series of voltammetric experiments by differential pulse cathodic stripping voltammetry (DPCSV) were carried out and they proved that the miniaturized sensor is characterized by very good accuracy and precision as well as long-term stability. The solid bismuth microelectrode displays a good voltammetric response in the analysis of diverse samples with a complex matrix and demonstrates a good recovery rate.

## 1. Introduction

Selenium is a natural component of the Earth’s crust, where it occurs in concentrations ranging from 5 × 10^−6^% [1] to 7 × 10^−5^% (m/m) [2,3] and that is why it is called a trace element. The circulation of selenium in nature begins with the weathering of rocks, from where it gets into groundwater and agricultural soils, and then this element is incorporated into the trophic chain of plants, animals, and humans. Selenium forms stable compounds in −2, +4, and +6 oxidation states [2]. Selenium comes in two forms: inorganic (selenites and selenates) and organic (Se-methionine and Se-cysteine). Both of them have been approved for animal and human nutrition [4].

Selenium is a necessary bioelement found in the human body in trace amounts, the presence of which determines the proper course of many physiological and biochemical processes. It occurs in enzymes and proteins. Selenoproteins play a valid role in reproduction, DNA synthesis, and protection against oxidation processes and infections [5,6]. It is an antioxidant which plays a beneficial role in limiting the toxic effects of xenobiotics, including heavy metals, as well as limiting the harmful processes of lipid, DNA, and RNA peroxidation [7,8,9]. Moreover, selenium can counteract some age-related changes as it contributes to increasing the elasticity of tissues, delays their aging, and alleviates menopausal symptoms [8]. It is involved in muscle metabolism. The lack of this element, especially in vitamin E deficiency, causes muscular dystrophy [10,11].

The daily requirement for selenium in Poland is set at 55 μg for women and 70 μg for men [11]. Despite a proper diet, many people suffer from deficiency of this element. It is a consequence of the increasing depletion of this element in the natural environment as a result of the use of modern technologies in agriculture and the food industry. The reduced supply is due to deepening soil erosion, acidification with sulfur and nitrogen compounds (“acid rain”), and contamination with heavy metals. The average selenium content in soils ranges from 0.1 to 2.0 mg/kg. In northern Europe, in the areas where glaciers reached, selenium was practically washed out of the soil. Poland also has soil poor in selenium [11,12]. Due to the above, the absorption of selenium by plants is inhibited. The limited transport of selenium along the food chain, from soil through plants and animals to humans, is manifested by its increasingly lower content in food products. The content of this element in meat and seafood is approximately 0.3–0.5 mg kg^−1^ [13]. In drinking water, the concentration of selenium is very low and ranges from 0.2 to 10 µg L^−1^ (on average 3.5 µg L^−1^); while in the waters in some parts of the world, the concentration of this element reaches up to 500 µg L^−1^ [14]. Natural mineral waters available in the Polish market contain selenium at an amount of 0.074–0.688 µg L^−1^, whereas the level of selenium in fruit juices ranges from 0.470 to 0.900 µg L^−1^ [15].

Due to the low concentration of selenium in the natural environment and, consequently, a low dietary intake of this element, people commonly struggle with selenium deficiencies. Selenium deficiency in humans may result in degeneration of many organs and organ tissues. The most well-known disease caused by the lack of this element is Keshan disease—an endemic heart muscle disease affecting mainly the heart muscle. Another one is Kashin-Beck endemic disease of the musculoskeletal system, causing ossification disorders and joint deformation. Furthermore, a relationship between selenium deficiency in the body and the reduced activity of enzymes responsible for the functioning of cell membranes, weakened immunity, and the development of cancer has been demonstrated [5,8]. The optimal selenium concentration for humans is 100–120 μg L^−1^ serum/plasma. A selenium concentration below 60 μg L^−1^ indicates a high risk of cancer. Unfortunately, this is the level found in 5% of Poland’s population, i.e., two million inhabitants. Additionally, it was proven that a low selenium concentration in Chinese residents correlated with the higher mortality of COVID-19 patients and a lower percentage of recoveries from this disease [11,16]. On the other hand, taking too high doses of selenium (i.e., above 400 micrograms per day) may contribute to the development of pulmonary edema, liver cirrhosis, cancer, and in extreme cases also death (which happens very rarely) [16].

On account of the beneficial effect of selenium on maintaining the body in health and, at the same time, due to its very low amounts in all sources from which selenium is taken by humans, there is a need to constantly monitor the level of this element in various environmental samples. Furthermore, the determination of such low selenium concentrations requires appropriately sensitive methods.

Electrochemical methods have played a significant role in trace element analysis not only because of their great sensitivity and selectivity, but also due to their easy handling, cost effectiveness as well as multielement and speciation capabilities. Amongst the electrochemical methods, stripping analysis is the most sensitive technique with detection limits in the ppb and sub-ppb range. This method makes possible the direct determination of Se(IV) (the only electrochemically active form). In order for total Se to be determined, reduction of Se(VI) to Se(IV) is necessary before analysis [17]. Our literature search has shown that stripping voltammetry has been extensively applied for the determination of selenium in various aqueous matrices and body fluids. In order to improve sensitivity and selectivity, the proposed voltammetric procedures for selenium determination used electrochemical sensors based on new materials enabling more effective preconcentration and determination of this component. Gold electrodes [17,18,19,20,21,22,23,24,25,26,27] and modified glassy carbon electrodes [28,29,30,31,32,33,34] are at the forefront of eco-friendly mercury-free electrodes most frequently used for selenium determination. In [25,26], gold electrodes were additionally modified with conducting polymers (o-Phenylenediamine-Nafion and Poly(3,30-diaminobenzidine), whereas gold nanoparticles [24,28,29,30], reduced graphene oxide [30,31], and a bismuth film [32,33,34] were used as modifiers of glassy carbon. Moreover, voltammetric procedures for selenium determination based on the use of a ceramic composite electrode modified with 2,3-diaminonaphthalene [35], a silver electrode [36,37], as well as a copper microelectrode [38] have also been developed.

In our research on selenium speciation, we focus on developing a procedure that would allow for the determination of the lowest concentrations of this microelement and could also be used for field analysis. The use of microelectrodes as a working electrode would provide such possibilities. To date, microelectrodes have been used to develop four procedures of selenium determination by stripping voltammetry [23,24,27,38]. In [23], arrays of gold microelectrodes were, for the first time, used to detect selenium, obtaining a lower detection limit (0.42 ppb) as compared with that reported for macroelectrodes. In [24], the application of two working electrodes significantly different in their surface area (a macroelectrode and an array of five gold microelectrodes), in conjunction with double deposition of selenium(IV), made it possible to decrease the detection limit of Se(IV) determined by anodic stripping voltammetry (ASV) and minimizing the interference effect. A gold microelectrode array was also used in [27], in which lowering the detection limit of selenium and at the same time slightly lowering the background current were achieved thanks to the use of double activation of the working microelectrode, at the beginning of the measurement and after the selenium accumulation stage. Single miniature electrodes with a cross-sectional diameter of less than 0.01 mm were also used in the speciation analysis of selenium by stripping voltammetry in [38], in which satisfactory reproducibility was obtained. Other work is based on the determination of selenium on macroelectrodes only [17,18,19,20,21,22,25,26,28,29,30,31,32,33,34,35,36,37]. Hence, it can be stated that there are very few voltammetric procedures for the determination of selenium developed using miniaturized sensors, which is surprising considering the fact that, currently, microelectrodes are gaining more and more practical importance due to their advantages. First of all, due to spherical diffusion around the area of the microelectrode, measurements can be made from unmixed solutions by using this group of working electrodes, which simplifies the measurement procedure and makes it possible to perform field analysis. Of course, a conventional electrode can also be used in the field, but in the case of this electrode, it is necessary to mix the sample during the accumulation stage. Small portable stirrers are possible to use for this purpose. Small dimensions of microelectrodes enable tiny volume samples to be analyzed. Moreover, the analyzed samples can be of organic origin as well as contain lower concentrations of the supporting electrolyte compared to the concentration necessary for measurements based on macroelectrodes [39]. A more favorable signal-to-noise ratio is another issue that distinguishes miniaturized sensors from larger ones, due to the reduction of capacitive current in these sensors to the minimum. All the above advantages are provided by the recently developed solid bismuth microelectrode that has so far been applied for the determination both of organic compounds (folic acid) [40] and inorganic ions (Tl(I), W(VI), Sn(II) and Ga(III)) [41,42,43,44] in different water samples. In these studies, the accumulation of the analyte to be determined was carried out directly on the solid bismuth microelectrode without bringing in any additional modifiers. As proven in this work, the increase in the sensitivity was achieved owing to the electrochemical-activated solid bismuth microelectrode, which usually lasts only 2 to 5 s. In addition, it features simplicity of preparation and easy handling as well as long-term stability and an eco-friendly character because a very small amount of metallic bismuth (tightly closed in miniaturized housing) is necessary to prepare this kind of electrode. Therefore, the application of a solid bismuth microelectrode does not require adding bismuth ions to the voltammetric cell, which makes the analysis environmentally friendly and greatly simplifies its execution. The use of this electrode for the determination of selenium would additionally have a certain advantage over the procedures already developed based on the use of bismuth film glassy carbon electrodes, in which it was necessary to add bismuth ions to the voltammetric vessel [32,33,34].

The challenge for our research was the use of a solid bismuth microelectrode to develop a voltammetric method for selenium determination, which would be the most sensitive among the already developed methods, based on the use of miniaturized sensors. To perform this, the differential pulse cathodic stripping voltammetry (DPCSV) method was used. The analytical usefulness of the elaborated method was confirmed by analyzing certified reference materials, such as SPS-SW1 (surface water) and TM 25.5 (lake water) as well as tap and mineral water.

## 2. Results and Discussion

### 2.1. Preliminary Studies

**Figure 1:**
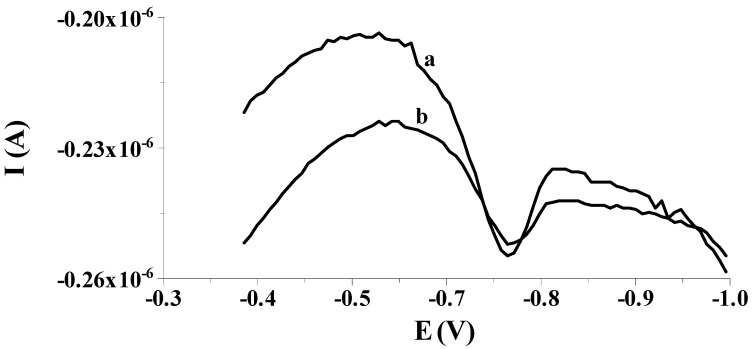
Exemplary voltammograms recorded for 8 × 10^−8^ mol L^−1^ Se(IV) used as a working electrode: solid bismuth ultramicroelectrode with activation at an activation potential of −2.5 V for 2 s (a); solid bismuth microelectrode without activation (b). Potential and time of accumulation: −0.550 V, 50 s; E (vs. Ag/AgCl, NaCl sat.); concentration of Se(IV): 8 × 10^−8^ mol L^−1^.

**Figure 2:**
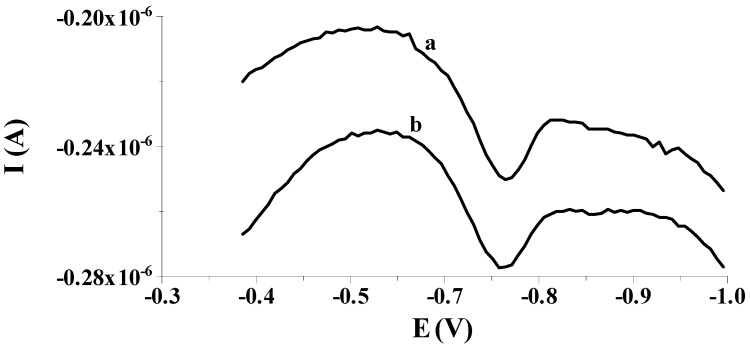
Exemplary voltammograms recorded during the course of Se(IV) determination from: (a) stirred solution during an accumulation step; (b) unstirred solution during an accumulation step. Potential and time of accumulation: −0.550 V, 50 s; E (vs. Ag/AgCl, NaCl sat.). Concentration of Se(IV): 8 × 10^−8^ mol L^−1^.

Our preliminary measurements showed that the previously proposed solid bismuth microelectrode [42,43,44,45,46] could be used to determine low selenium concentrations by differential pulse cathodic stripping voltammetry (DPCSV) in aqueous samples under field conditions. As in previous works, the working microelectrode was subjected to a short electrochemical activation process at a potential of −2.5 V for 2 s, during which, as mentioned earlier, bismuth oxides that may form on the electrode surface are reduced to a metallic form. Owing to the application of the activation process, better shaped and much higher analytical signals of selenium were acquired compared to the signals acquired without the activation stage, as shown in Figure 1. Moreover, the application of the activation stage makes the results acquired much more repeatable. The obtained results are promising and give hope for the development of a more sensitive procedure compared to those already developed using microelectrodes. Then, in order to check whether the electrode would work well in field analysis, the accumulation of selenium on the solid bismuth microelectrode was performed without mixing the sample solution and the obtained results were compared to those gained when using the preconcentration step from a mixed solution. Figure 2 shows a comparison of both peaks. The obtained results confirm that due to spherical diffusion occurring in the microelectrode, there is no need to mix the solution during the preconcentration stage as well; as such, a sensor can be successfully used to analyze the sample at the point of its collection. Therefore, a series of further experiments were carried out to optimize the chemical and instrumental parameters affecting the measured selenium signal. The chemical variables were the composition of the supporting electrolyte and its pH, whereas the instrumental variables examined were the potential and time, both of activation of the microelectrode surface and accumulation of selenium. All optimization studies were conducted for a synthetic sample with a selenium concentration of 5 × 10^−8^ mol L^−1^. The synthetic sample contained Se(IV) in the form of H_2_SeO_3_.

### 2.2. Optimization Studies

#### 2.2.1. The Effect of Supporting Electrolyte Composition

**Figure 3:**
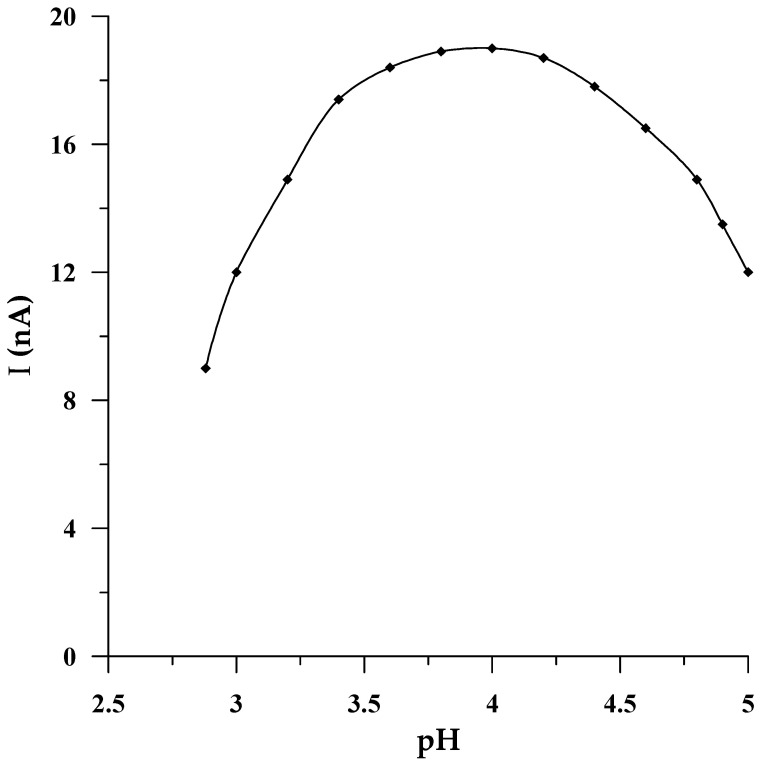
The impact of pH of supporting electrolyte on the 5 × 10^−8^ mol L^−1^ Se(IV) peak current.

In all procedures in which a solid bismuth microelectrode was used as a working electrode, the best voltammetric response of this electrode was achieved in an acidic medium, provided mainly by acetate buffers of various pHs, most frequently a pH equal to 4.6 [42,43,45], and in one case, acetic acid [43]. The impact of pH on the microelectrode performance in the current work was consequently studied by using acetic acid (pH = 2.88) as an initial solution, whose pH was gradually increased to 5.0 by putting in place aliquots of NaOH (2 mol L^−1^) to the voltammetric cell. Figure 3 shows the change in the Se(IV) peak current intensity as a function of pH. The presented dependence demonstrates that the electrode produces the most favorable selenium signal in the medium of the acetate buffer pH = 4.0 ± 0.1, and conversely, both for lower and higher pH values, the downward trend in the voltammetric signal intensity is noticeable. The obtained dependence may be related to the chemical form in which Se(IV) occurs in the tested pH range. In the synthetic sample, selenium(IV) occurs in the form of weak selenic acid, which undergoes the following reversible reaction: H_2_SeO_3_ ⇌ SeO_2_ + H_2_O. Therefore, it can be assumed that at pH = 4.0 ± 0.1 the equilibrium between H_2_SeO_3_ and SeO_2_ in the tested solution is established. Whereas at lower and higher pH, the equilibrium shifts towards the formation of oxide or acid, respectively. Considering that at pH 4.0, an acetate buffer provides the best buffer capacity, an acetate buffer with this pH was selected to obtain an adequate analytical environment for subsequent measurements. The next stage of the research focused on determining the relationship between the electrode sensitivity and the concentration of the buffer used as a supporting electrolyte. It was found that despite the change in the concentration of the supporting electrolyte in the range from 0.05 to 0.2 mol L^−1^, the electrode sensitivity remained unchanged. Therefore, 0.1 mol L^−1^ CH_3_COOH/CH_3_COONa at pH = 4.0 was applied for all the studies.

#### 2.2.2. The Effect of Activation Conditions on the Electrode Performance

**Figure 4:**
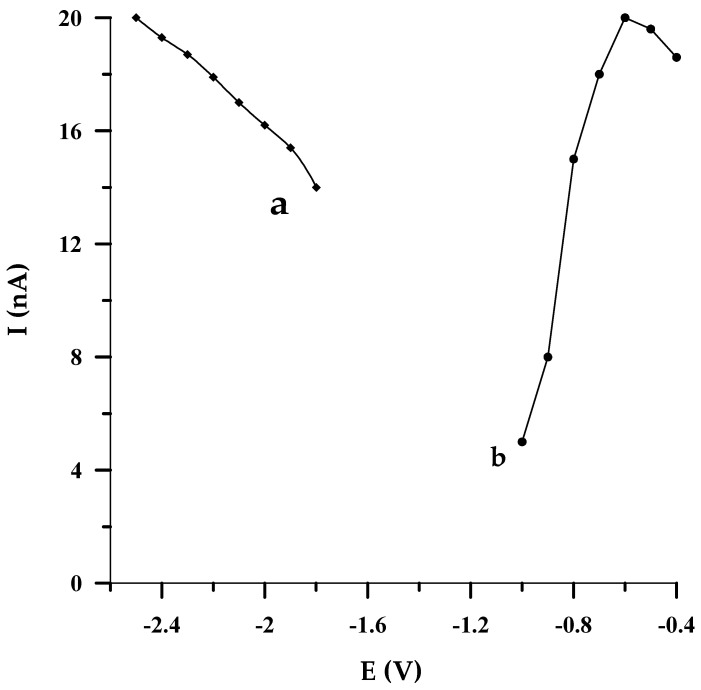
The impact of activation potential (a) and accumulation potential (b) on 5 × 10^−8^ mol L^−1^ Se(IV) peak current; E (V vs. Ag/AgCl, NaCl sat.).

In the proposed DPCSV method, the potential of electrode activation was chosen by altering it in the range from −2.5 to −1.8 V, every 0.1 V (vs. Ag/AgCl, NaCl sat.). Each studied potential value was applied to the electrode for 2 s. As is evident in Figure 4a, when the potential value changed towards a less negative one, the electrode response decreased slightly. In conformity with the acquired data, the activation potential of −2.5 V (vs. Ag/AgCl, NaCl sat.) was taken as the most optimal for further voltammetric experiments.

Then, we focused on picking out the most favorable activation time. To this end, an activation potential of −2.5 V (vs. Ag/AgCl, NaCl sat.) was applied to the electrode at a time interval from 1 to 7 s. The tests performed revealed that irrespective of the duration of the electrode activation process, the peak intensity, its shape, and position on the voltammogram were invariable. Therefore, in order not to unnecessarily prolong the duration of a single measurement, it was decided to activate the electrode in subsequent measurements by applying a potential of −2.5 V (vs. Ag/AgCl, NaCl sat.) for 2 s.

#### 2.2.3. The Effect of Accumulation Conditions on the Electrode Performance

By optimizing the potential at which selenium accumulated on the solid bismuth microelectrode, it turned out that the DPCSV response of the sensor was improved when the potential of Se(IV) accumulation was varied from −400 mV to −600 mV (vs. Ag/AgCl, NaCl sat.). Afterwards, the more negative the potential that was applied to the electrode, a larger depression of the analytical signal was observed (Figure 4b). Because the best peak shape was obtained at the accumulation potential of −550 mV (vs. Ag/AgCl, NaCl sat.), this value was acknowledged as the optimal one. It should also be emphasized that owing to the radial diffusion, greater accumulation may be achieved on a microelectrode than on a conventional electrode at the same potentials.

At the same time, by accumulating selenium on the solid bismuth microelectrode at different periods of times (from 10 to 100 s), it proved that the peak current first exhibited an upward trend and then attained the maximum upon extending the accumulation time to 50 s. A further extension of the accumulation time does not affect the signal intensity, which remains constant. Hence, in this DPCSV procedure, selenium accumulates on the working microelectrode at a potential of −0.550 V (vs. Ag/AgCl, NaCl sat.) for 50 s.

### 2.3. Analytical Figures of Merit

**Figure 5:**
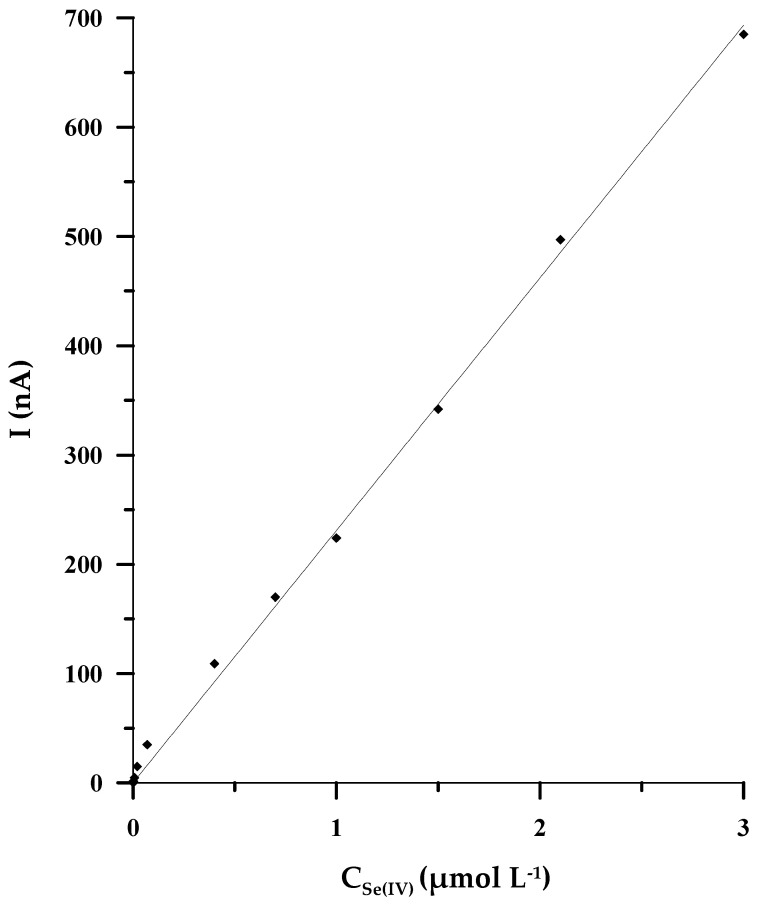
The calibration curve for the solution 0.1 mol L^−1^ CH_3_COOH/CH_3_COONa (pH = 4.0), and changeable concentrations of Se(IV) in the scope from 2 × 10^−9^ mol L^−1^ to 3 × 10^−6^ mol L^−1^.

Under the optimal experimental circumstances, both chemical and instrumental ones, a linear correlation between the intensity of the analytical signal and the concentration of Se(IV) was found in the concentration range of 2 × 10^−9^ to 3 × 10^−6^ mol L^−1^. The equation of the linear regression is y = (231 ± 5)x, coefficient of determination R^2^ = 0.9988 (x: concentration/μmol L^−1^, y: peak’s current/nA) (Figure 5). The validation variables, such as detection limit (LOD) and quantification limit (LOQ), were appraised based on the analysis of the calibration curve. The LOD and LOQ were calculated from the following equations: LOD = 3 × SD/b and LOQ = 10 × SD/b (SD is defined as the standard deviation of the signal blank; b is the slope of the calibration curve), and they were equal to 5 × 10^−10^ mol L^−1^ and 1.7 × 10^−9^ mol L^−1^, respectively. The analytical features of the described approach and the other stripping voltammetric methods developed based on the use of microelectrodes and bismuth film glassy carbon electrodes are presented in Table 1. Among all the compared methods, the DPCSV procedure proposed in the current paper exhibits the widest range of linearity, encompassing three orders of magnitude, such as in [32], but slightly lower selenium concentrations, equal to 2 nmol L^−1^, can be determined by using our procedure. At the same time, the procedure we have developed shows the highest sensitivity of all voltammetric methods based on the use of microelectrodes. Apart from that, the duration of the accumulation stage is relatively short, lasting only 50 s.

### 2.4. Stability of Measurements

To investigate the repeatability of the solid bismuth microelectrode, the Se(IV) peak current was successively recorded seven times, for two various selenium concentrations, notably 4.0 nmol L^−1^ and 0.2 µmol L^−1^, and RSDs equal to 4.2 and 2.8%, respectively, were obtained. The reproducibility of the electrode was also determined based on measurements taken over seven consecutive days for a concentration of 2.0 µmol L^−1^ and the RSD was calculated as 4.8%. The long-term stability was determined after two and six months of use of the electrode and it was found that regardless of whether the signal was recorded after two or six months, the peak height changes did not exceed ±5% compared to the signal recorded at the beginning of its use. Our experimental data show that the accuracy of the method is satisfactory and the solid bismuth microelectrode exhibits very good precision (see Section 2.6. *Accuracy and analytical usefulness*) and has long-term stability.

### 2.5. Tolerance to Interfering Factors

The selectivity of the solid bismuth microelectrode to Se(IV) was examined in the presence of miscellaneous metal ions and Triton X-100. As has been proven in research, Triton X-100 occurring in a voltammetric cell at the concentration range from 0.2 to 2.0 mg L^−1^ has a similar impact on the analytical signal as surfactants in natural waters [45]. Hence, the impact of 2.0 mg L^−1^ of Triton X-100 on the 1.0 × 10^−8^ mol L^−1^ Se(IV) voltammetric signal was studied and turned out to be irrelevant. As for the influence of other cations, it was found that at least a 100-fold excess of Co(II), Cr(III), Cr(VI), Fe(III), Ga(III), Ge(IV), Hg(II), Mn(II), Ni(II), Pb(II), Pt(IV), Sb(III), Sn(II), V(V), and Zn(II) is tolerable within an error of ±5%, while a 10-fold excess of Cd(II) and Cu(II) causes the selenium peak to be reduced by 50%. As previously proven, the presence of one of the interfering metals, such as cadmium or copper, in a solution containing selenium causes disturbing effects that result from a reaction between cadmium/copper and selenium, producing the formation of alloys or an intermetallic compound in the course of the accumulation stage on the electrode surface [18,31,34,46]. Nevertheless, the addition of 2 × 10^−4^ mol L^−1^ EDTA to the tested solution utterly eliminated the interference generated by Cd(II) and made it possible to determine selenium in the presence of 2 × 10^−7^ mol L^−1^ Cu(II), as in [32].

### 2.6. Accuracy and Analytical Usefulness

**Figure 6:**
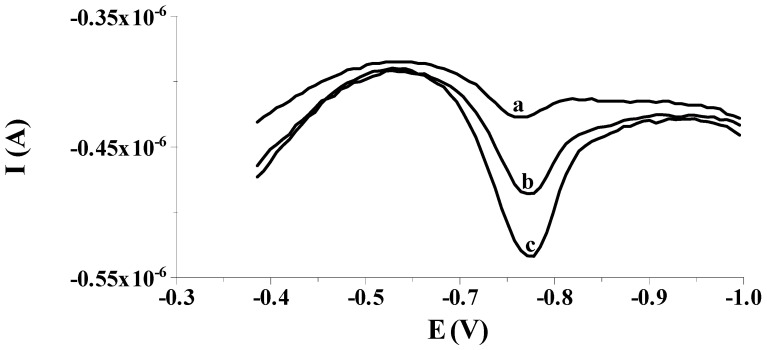
The DPCSV voltammogram responses made in the course of selenium analysis in the certified reference material TM-25.5: TM-25.5 diluted 10-fold (a), as (a) + 0.15 µmol L^−1^ Se(IV) (b); as (a) + 0.30 µmol L^−1^ Se(IV) (c). All voltammograms were obtained in the following conditions: activation of the electrode at a potential of −2.5 V for 2 s; accumulation of selenium at a potential of −0.550 V for 50 s, E (vs. Ag/AgCl, NaCl sat.); composition of the supporting electrolyte: 0.1 mol L^−1^ acetate buffer pH = 4.0.

The analytical usefulness of the proposed method was assessed by determining Se(IV) in two certified reference materials (SPS-SW1 and TM-25.5); the composition of the matrix and the concentration of metal ions in either of the CRMs are different. The CRMs were introduced into a voltammetric vessel with an appropriate amount of 1 mol L^−1^ NaOH (due to the content of nitric acid in the tested materials) and were diluted with 0.1 mol L^−1^ acetate buffer. Afterwards, they were analyzed using the standard addition method and three voltammograms were recorded for each CRM and each standard add-on. The analytical results of Se(IV) determination obtained for SPS-SW1 (2.09 ± 0.14 µg L^−1^) and TM-25.5 (28.1 ± 3.3 µg L^−1^), were consistent with the certified values (2.00 ± 0.02 µg L^−1^ and 29.2 ± 3.5 µg L^−1^ for SPS-SW1 and TM-25.5, respectively) (Table 2). Furthermore, the developed method was employed to determine Se(IV) in tap and mineral water, but in neither of these samples was Se(IV) determined via the described method. To assess the applicability of the procedure in these types of matrices, these water samples were enriched with different concentrations of Se(IV) and then they were left for 1 h to equilibrate. Finally, each of the enriched samples was tested 10 times after dilution with 0.1 mol L^−1^ acetate buffer, one by one, as previously mentioned. The data depicted in Table 3 point out that all of the tested samples do not contain a selenium concentration detectable by our procedure and the recoveries calculated for the samples enriched with various amounts of Se(IV) are between 91.5% and 97.4% with the RSD ranging from 4.4% to 6.1%. The results collated in Table 2 and Table 3 confirm that the described voltammetric approach using the solid bismuth microelectrode is an adequate approach to determination of Se(IV) in real samples containing a complex matrix and shows satisfactory accuracy. Figure 6 shows exemplary voltammograms recorded during the detection of Se(IV) in the certified reference material TM-25.5.

## 3. Materials and Experimental Work

### 3.1. Apparatus and Instruments

All electrochemical investigations were carried out with a µAutolab (Eco Chemie, Utrecht, The Netherlands) connected via a USB connection to a personal computer installed with the GPES 4.9 measurement software package. A three-electrode cell (10 mL) was utilized, with a solid bismuth microelectrode (Ø = 25 µm) as the working electrode, an Ag/AgCl (with saturated NaCl) electrode as the reference electrode, and a platinum wire electrode as the counter electrode. All potentials were measured and reported vs. the Ag/AgCl (NaCl sat.) electrode. An itemized delineation of the solid bismuth microelectrode design and its first usage are depicted in [40]. Every day prior to its use, the working microelectrode was polished on 2500 grit sandpaper, subsequently rinsed thoroughly with copious amounts of deionized water, and kept in an ultrasonic bath for 30 s to dislodge any residual polishing material. An Orion Star A211 pH benchtop meter (Thermo Scientific, Waltham, MA, USA) was used to measure pH values of the solutions.

### 3.2. Chemicals

Acetate buffers were prepared from acetic acid and sodium hydroxide (Suprapur, Merck, Warsaw, Poland). A stock solution of 1 g L^−1^ Se(IV) was obtained from Sigma Aldrich. This solution contains Se(IV) in the form of SeO_2_ in HNO_3_ (H_2_SeO_3_). The standard solution of Se(IV) and other ions was prepared by dissolving an appropriate amount of their nitrate or chloride with deionized water. All solutions were prepared from deionized water produced by a water purification Milli-Q system (Millipore, London, UK) and analytical grade or Suprapur reagents. For the validation of the procedure, certified reference materials, such as SPS-SW1 (surface waters, Spectrapure Standards, Oslo, Norway) and TM-25.5 (Lake Ontario water, Environment and Climate Change, Toronto, Ohio, Canada), were used.

### 3.3. Measurement Procedure

The bismuth microelectrode (BiµE) was placed in 10 mL of a 0.1 mol L^−1^ acetate buffer (pH = 4.0) containing a certain amount of Se(IV) and activated by applying an activation potential of −2.5 V (vs. Ag/AgCl, NaCl sat.) for 2 s (during this stage, bismuth oxides that may form on the electrode surface are reduced to a metallic form according to the chemical reaction Bi_2_O_3_ + 6H^+^ + 6e− → 2Bi^0^ + 3H_2_O), followed by selenium accumulation at an electrolysis potential of −550 mV (vs. Ag/AgCl, NaCl sat.) for 50 s (during this stage, selenium (IV) is reduced to selenium (0)). In many studies, it was considered that at a low reduction potential of accumulation (in the range of −0.2 to −0.4 V or even more electronegative potential), the reduction in Se(IV) takes place with the participation of six electrons, resulting in the formation of H_2_Se, in accordance with the reaction: H_2_SeO_3_ + 6H^+^ + 6e_−_ → H_2_Se + 3H_2_O. Moreover H_2_Se, in acid solution, is known to undergo a comproportionation reaction in the presence of selenous acid, as follows: H_2_SeO_3_ + 2H_2_Se → 3Se + 3H_2_O [27,28,31,37]. Next, for the stripping step, after 5 s of the rest period, the BiµE was polarized in the range of potentials from −400 mV to −1000 mV (vs. Ag/AgCl, NaCl sat.) and the DP voltammogram was registered. The stripping step is probably based on the reduction of Se^0^ to Se^2−^. Thanks to the reduction process, the registration of a well-shaped peak at a potential of −0.75 V takes place, which is the basis for the quantitative analysis of Se(IV) in the proposed procedure. In this step, the stirrer was switched off and the step potential, modulation amplitude, and scan rate were 0.005 V, 0.025 V, and 10 mV s^−1^, respectively. All electrochemical measurements were performed at room temperature (22 ± 1.5 °C) without deletion of oxygen from the solution.

## 4. Conclusions

The current work proposes the application of a solid bismuth microelectrode for the determination of Se(IV) by the differential pulse cathodic stripping voltammetry (DPCSV) method in environmental water samples with a complex matrix at the point of their collection. Owing to the use of a solid bismuth microelectrode elaborated by deposition of a very thin amount of metallic bismuth in a solid Bi electrode tightly closed in miniaturized housing, the method developed in this work is the most sensitive among those based on the use of miniaturized sensors. The design of the electrode makes it more friendly to the laboratory environment than the previously widely used bismuth film electrodes. The amount of metallic bismuth needed for fabrication of the proposed sensor is significantly minimized as compared to the solid bismuth electrode with a large surface area. Moreover, in comparison to bismuth film electrodes, bismuth ions are not added to the supporting electrolyte for bismuth film formation. Moreover, the innovative solid bismuth microelectrode enables simple and quick detection of Se(IV) without the need to use a large number of reagents as only the supporting electrolyte is needed, while the total time of a single measurement is less than a minute. The research carried out in this work proves that this miniaturized sensor ensures excellent precision, which resulted in obtaining an extremely repetitive and reproducible voltammetric response during the analysis of real samples (certified reference materials, such as SPS-SW1 (surface water) and TM 25.5 (lake water) as well as tap and mineral water) and a good recovery rate for those samples in which the selenium level was below that determined by this method. Furthermore, the sensor is characterized by long-term stability, since it has been shown that after six months of use, this microelectrode still maintains signal stability within ±5%, as at the beginning of its use.

## Figures and Tables

**Table 1:** An overview of the voltammetric procedures for Se(IV) determination developed based on the use of microelectrodes as well as bismuth film glassy carbon electrodes. The methods are ranked according to increasing limit of detection.

Electrode	Method	LOD (nmol L^−1^)	Linear Range (nmol L^−1^)	Accumulation Time (s)	Application	Ref.
BiF/GCE	DPAdSV	0.63	25–633	90	blood and urine samples	[33]
BiµE	DPSV	0.70	2–3000	50	SPS-SW1 and TM-25.5 CRMs	This work
BiF/GCE	SWASV	0.80	3–3000	65	SPS-SW1 and TM-25.5 CRMs, river, tap, mineral and rain water	[32]
AuµEs	SWASV	0.83	3–30	180	SPS-SW1 and TM-25.5 CRMs	[27]
AuµEs and AuE	SWASV	0.85	5–100	300	SPS-SW1 CRM	[24]
BiF/GCE	DPAdSV	1.30	25–380	300	real water samples	[34]
Au UMEs	SWASV	5.30	10–1266.5	-	-	[23]
CuµE	SWCSV	-	5000–50,000	15	-	[38]

BiF/GCE—bismuth film glassy carbon electrode; AuµEs—array of gold microelectrodes; AuE—gold electrode; Au UMEs—array of gold ultramicroelectrodes; CuµE—copper microelectrode.

**Table 2:** Results obtained during the Se(IV) determination in the certified reference materials.

Certified Reference Material	Experimental Value ± SD (*n* = 3) [µg L^−1^]	Certified Value ± SD (*n* = 3) [µg L^−1^]
SPS SW-1	2.16 ± 0.14	2.00 ± 0.02
TM-25.5	28.1 ± 3.3	29.2 ± 3.5

**Table 3:** Recovery values for Se(IV) acquired from spiked water samples by means of the described DPCSV procedure.

Sample	Se(IV) Added [nmol L^−1^]	Se(IV) Determined [nmol L^−1^]	Recovery [%]	RSD (*n* = 3) [%]
Tap water	0.0	0.0	-	-
	50.0	48.7	97.4	4.4
	100.0	91.5	91.5	6.8
Mineral water	0.0	0.0	-	-
	50.0	46.3	92.6	5.2
	100.0	93.9	93.9	6.1

## Data Availability

Data are contained within the article.
